# *MAIN* software for density averaging, model building, structure refinement and validation

**DOI:** 10.1107/S0907444913008408

**Published:** 2013-06-13

**Authors:** Dušan Turk

**Affiliations:** a Biochemistry and Molecular and Structural Biology, Jozef Stefan Institute, Jamova 39, Ljubljana, Slovenia; b Centre of Excellence for Integrated Approaches in Chemistry and Biology of Proteins, Jozef Stefan Institute, Jamova 39, SI-1000 Ljubljana, Slovenia

**Keywords:** molecular modelling, molecular graphics, macromolecular crystal structure determination, map calculation, computer programs

## Abstract

*MAIN* is software that has been designed to interactively perform the complex tasks of macromolecular crystal structure determination and validation. Using *MAIN*, it is possible to perform density modification, manual and semi-automated or automated model building and rebuilding, real- and reciprocal-space structure optimization and refinement, map calculations and various types of molecular structure validation. The prompt availability of various analytical tools and the immediate visualization of molecular and map objects allow a user to efficiently progress towards the completed refined structure. The extraordinary depth perception of molecular objects in three dimensions that is provided by *MAIN* is achieved by the clarity and contrast of colours and the smooth rotation of the displayed objects. *MAIN* allows simultaneous work on several molecular models and various crystal forms. The strength of *MAIN* lies in its manipulation of averaged density maps and molecular models when noncrystallographic symmetry (NCS) is present. Using *MAIN*, it is possible to optimize NCS parameters and envelopes and to refine the structure in single or multiple crystal forms.

## Abstract 2

*MAIN* is interactive software designed to interactively perform the complex tasks of macromolecular crystal structure determination and validation. The features of *MAIN* and its tools for electron-density map calculations, model building, refinement in real and reciprocal space, and validation exploiting noncrystallographic symmetry in single and multiple crystal forms are presented.

## 1. Introduction

Shortly after my arrival at the Max Planck Institute for Biochemistry in Martinsried in 1988, I began working in crystal structure determination. I realised that the variety of different programs and specific environments created a serious hindrance to an effective macromolecular structure-determination process. Therefore, I decided to expand *MAIN* from molecular-graphics software (Turk, 1988 ▶) into general crystal structure-determination software, integrating a variety of tools into a single working environment. Initially, the development of *MAIN* was driven by the demands of the projects that the group was involved in. This work ultimately led to my PhD thesis (Turk, 1992 ▶). Subsequent *MAIN* develop­ments also remained demand-driven; however, over the years it became a research project in which tools were appended to explore concepts and ideas, tackling various aspects of structure determination. *MAIN* developments have mostly been presented at conferences and provided to the users, so that the concepts and ideas could find their way into the community; however, the basic citation remained my PhD thesis (Turk, 1992 ▶).

Macromolecular crystal structure determination is a complex process involving a number of steps. However, after the molecule of interest has been crystallized and the diffraction data have been collected, crystal structure determination becomes a computational procedure. After solving the phase problem, *MAIN* can be used to

(i) display molecular models and electron-density maps,
(ii) calculate various types of electron-density maps,
(iii) build models from scratch and extend and rebuild existing models using manual, semi-automated and automated tools in combination with real-space energy minimization,
(iv) perform interactive structure validation, and
(v) refine structures against real-space and reciprocal-space targets.

The development of the features of *MAIN* occurred hand in hand with the progress of other software and interfaces to them. Obviously, a number of tools and areas have remained outside the scope of the development of *MAIN*. In this paper, the features and tools of *MAIN* are described by looking back at the projects that led to their birth, followed by two recent cases and the foundations of three-dimensional perceptions of interactive graphics and performance.

In the last decade, automatic building of initial models has made substantial progress and its use is widely accepted in the crystallographic community. The development of programs such as *ARP*/*wARP* (Langer *et al.*, 2008 ▶), *SOLVE*/*RESOLVE* (Terwilliger & Berendzen, 1999 ▶), *Buccaneer* (Cowtan, 2006 ▶), the *SHELX* group of software (Sheldrick, 2010 ▶) and other programs are providing increasingly complete models in increasingly integrated environments such as *CCP*4 (Winn *et al.*, 2011 ▶), *PHENIX* (Adams *et al.*, 2010 ▶) and *HKL*-3000 (Minor *et al.*, 2006 ▶).

However, molecular models must be completed, rebuilt and extended. *MAIN* offers a number of features and tools that perform many of the tasks of crystal structure determination. The interface and tools are somewhat different from those present in programs such as *Coot* (Emsley *et al.*, 2010 ▶) and *O* (Jones & Kjeldgaard, 1997 ▶). In particular, at low resolution there are cases in which manual intervention in model building must begin quite early, either to extend and remove misplaced regions of automatically generated models or to begin from scratch. *MAIN* is especially suited for handling large structures and those with multiple subunits owing to its display capabilities and electron-density manipulation and model-building tools, combined with features that address non­crystallographic symmetry (NCS) issues, as demonstrated in cases such as lumazine synthase (Ritsert *et al.*, 1995 ▶), tetrahydropterin synthase (Nar *et al.*, 1994 ▶), GTP hydrolase I (Nar *et al.*, 1995 ▶) and proteasomes (Groll *et al.*, 1997 ▶; Löwe *et al.*, 1995 ▶) which were essential in the development of *MAIN*.

Below, a description of the areas of structure determination included in *MAIN* is presented. It is followed by the description of two recent rather typical cases chosen to demonstrate the uses of *MAIN*. They both exploit fourfold NCS. The first case is based on selenomethionine phasing at a resolution below 3 Å, whereas the second case uses molecular replacement of a partial structure. Finally, an insight into the design of *MAIN* and its current performance is provided. In the Supplementary Material^1^, a brief outline of the user interface is presented.

## 2. Areas of structure determination included in *MAIN*

**Figure 1:**
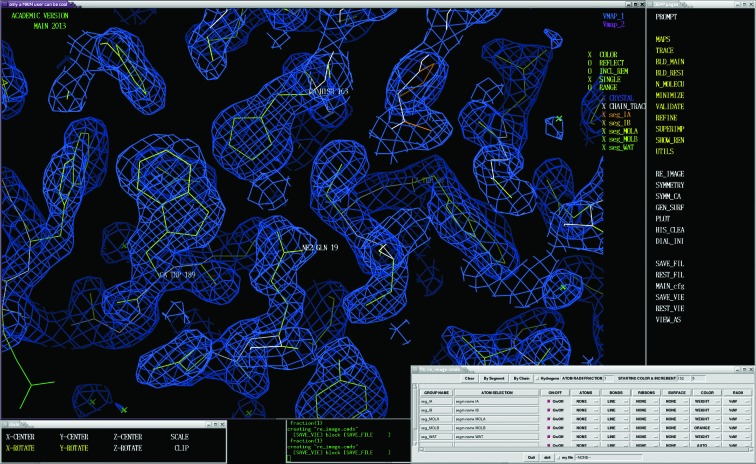
Snapshot of a *MAIN* working session. On the top left is the large ‘only a *MAIN* user can be cool’ window. The ‘DEPP pages’ menu window is on the left. At the bottom on the top right is the ‘dials’ window describing the functionality of the dial box and mouse. At the bottom right corner is the ‘Tk_re_image.cmds’ window laying out the GUI interface to the molecular-image composition.

The areas of structure determination included in *MAIN* are organized in the topical page items as can be seen on the right of the *MAIN* working-session screen shown in Fig. 1 ▶. (A brief user interface is described in the Supplementary Material.) When clicked, the topical page item rebuilds the menu page by displaying the topical page menu blocks and their items (Table 1 ▶). The topical pages are the following.

(i) The MAPS page addresses map calculation, manipulation and display.
(ii) The TRACE page addresses automated initial model building. This page remains a work in progress and is therefore not described here.
(iii) The BLD_MAIN and BLD_RESI pages provide an interface to manual and semi-automated model-building tools that cover initial model building, model extension and rebuilding. The BLD_RESI page also contains the validation tools.
(iv) The N_MOLECU page addresses the NCS manipulation of molecules, enabling creation of the molecular model and exchanges between the different related subunits.
(v) The MINIMIZE page provides the interface to energy minimization, hydrogen-bonding restraint-list creation and manipulation, and secondary-structure recognition and assignment.
(vi) The REFINE page addresses structure refinement, including solvent generation.
(vii) The SUPERIMP page addresses the superimposition of molecular models.
(viii) The MAP_MASK page addresses the map scoring function, molecular-map manipulation and skeleton creation and editing to assign the asymmetric unit.

### 2.1. Maps

**Figure 2:**
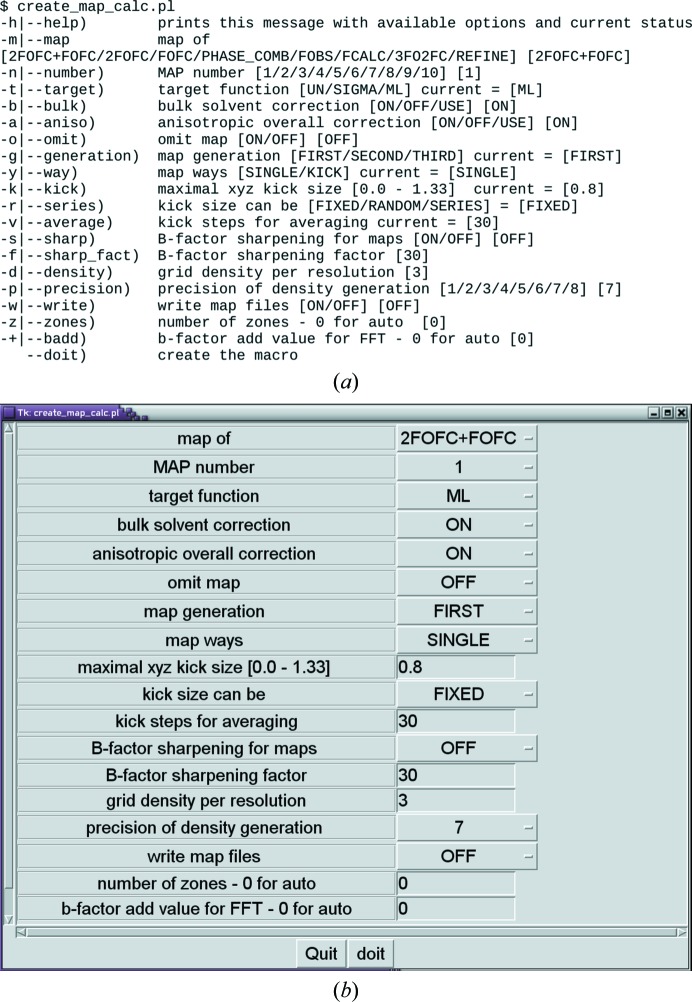
Map-calculation setup. (*a*) Text version. (*b*) Tk-GUI translation of the text version. The Tk-GUI form is compiled from the text version by submitting the create_map_calc.pl script without any parameters provided. In the help for the text version multiple choices are provided in parentheses. In the Tk interactive user interface these choices are provided as option menus.

In *MAIN*, electron-density maps can be calculated on the fly from molecular models and structure-factor files or read from a file. The map calculations are configured *via* the GUI interface macro (create_map_calc.pl; MAP_CALC; Fig. 2 ▶). There are numerous options and possibilities. For example, one can calculate maps from imported structure factors (*F*_obs_), perform phase combination by accessing a program such as *SIGMAA* (Read, 1986 ▶) and calculate difference maps from molecular models, such as maximum-likelihood weighted (Lunin *et al.*, 2002 ▶), unweighted or kick maps (Pražnikar *et al.*, 2009 ▶). In map calculation from molecular models, bulk-solvent correction using molecular envelopes (Fokine & Urzhumtsev, 2002 ▶) and six-parameter anisotropic *B*-factor correction are used. *B*-value sharpening of lower resolution maps can also be applied. Parts of models can be omitted either by changing the occupancy of the atoms or by selecting regions for their omission.

To calculate less model-biased maps, the approach termed kick maps is used (Pražnikar *et al.*, 2009 ▶). Kick maps are based on the idea that independent random shifts introduced into atomic coordinates would disrupt the correlations of atomic positions imposed by refinement through their interactions with the structure factors and the chemistry terms. As a result, random shifts of coordinates, termed kicking, was introduced into *MAIN* (Guncar *et al.*, 1998 ▶; Turk, 1997 ▶, 2007 ▶) and applied in map calculations and refinement. Averaging the individual kick maps indicated that they have the potential to reduce or eliminate model bias. The resulting procedure appears to be analogous to the maximum-likelihood approach: the crystallographic maximum-likelihood theory supposes that the current model can be corrected by introducing random errors and suggests the correction of the structure factors after ‘theor­etical averaging’ of such models with random shifts. This procedure can be a useful alternative scheme for map calculations at any stage of structure solution (Pražnikar *et al.*, 2009 ▶). It has been demonstrated that these maps are an improvement over single kick maps (Guncar *et al.*, 2000 ▶; Than *et al.*, 2002 ▶, 2005 ▶). Additionally, the averaged kick maps revealed the presence of active and inactive conformations in the α-tryptase structure (Rohr *et al.*, 2006 ▶). Moreover, the concept of second-generation averaged kick maps revealed that these maps can be used to remove inconsistent regions from phasing and thereby reduce model bias at a slight cost in map clarity (Pražnikar *et al.*, 2009 ▶). In the same work it was also shown that averaged kick maps can offer advantages over other maps such as unweighted, maximum-likelihood weighted and simulated-annealing maps.

For the storage of data and their exchange between various programs, the use of structure-factor files is recommended over electron-density map files. Reading of a structure-factor file followed by an internal fast Fourier transformation calculation (Ten Eyck, 1977 ▶) is significantly faster than reading large electron-density map files. Additionally, in *MAIN* maps are calculated for the entire unit cell and can therefore be displayed anywhere in space using the *P*1 symmetry operator.

#### 2.1.1. Map averaging: general

Map averaging was the first density-manipulation procedure to be written in *MAIN*. The human and rat cathepsin B proteins (Musil *et al.*, 1991 ▶) were the first molecules to be averaged. Next, we tackled lumazine synthase crystals in the monoclinic form (Ritsert *et al.*, 1995 ▶). At the time this was a massive project, with 50 000 atoms and 30 molecules in the asymmetric unit. Phase extension from 10 Å to full resolution (2.3 Å) took approximately half a year on a dedicated MicroVAX computer. Currently, a cycle of a similar averaging procedure for a molecule of comparable size, such as the proteasome (Groll *et al.*, 2006 ▶), takes less than a minute.

Averaging in *MAIN* can be performed between any number of equivalent regions and any number of different crystal forms in any possible combination. A user does not have to worry about the particular asymmetric unit definition, as the unit-cell generation procedure maps each grid point to its symmetry-equivalent point by applying the symmetry operators. However, there is a requirement that the equivalent regions are contiguous areas in space and that they are not larger than the unit cell in any direction.

To perform real-space electron-density averaging, an initial set of phases, molecular masks (also termed envelopes) and superimposition operators (rotation and translation parameters) are required. *MAIN* uses segment identifiers (segment names) to assign local subunits and corresponding masks and the superimposition parameters between them.

#### 2.1.2. Map averaging: molecular-mask creation

When performing the density-modification procedure, its basis is the molecular mask (sometimes termed the molecular envelope), which demarcates the macromolecule from the solvent region. In *MAIN*, the mask is a map data structure. The difference between the density and the mask region is the value at the grid point, which must be greater than 9990 for the masked regions.

Before masks are created, it is important to assign the type of NCS to the case. There are two types of NCS: proper (also called spherical) and improper (Rossmann & Blow, 1962 ▶). The molecules in an asymmetric unit are related by proper symmetry when they can be superimposed on each other by rotation(s) about the centre only, whereas in the case of improper symmetry the operators of superimposition include a translational component in addition to the rotational component. Correspondingly, the procedures for averaging density with proper and improper NCS also differ. For proper symmetry averaging the entire group of molecules is enclosed within a single mask, whereas for improper averaging separate masks must be generated for each subunit. *MAIN* uses the keywords WHOLE for proper and EACH and ONE for improper symmetry cases. (These keywords should be specified even when no atomic model is available). Occasionally, proper and improper symmetries are combined and can be layered. An improper NCS symmetry unit may be composed of subunits arranged with proper symmetry. However, layering requires the users to edit their macros. In *MAIN*, density can also be averaged between a variety of crystal forms. (Four are supported by the GUI; to average more either a manual intervention into the macros or recompilation of the source code is required.)

The simplest way to create a mask is from an atomic model. Atomic models can represent a macromolecular structure, positions of heavy-atom derivatives or a skeleton derived from an electron-density map. The volume occupied by the atomic model is defined by the positions of the atoms and their size. The default starting atomic radii are set to 6 Å. To avoid overlaps between atoms from different groups related by NCS or crystallographic symmetry, the atomic radii are reduced to half of the closest interatomic distance. At the beginning, when, for example, the molecular model is represented only by heavy atoms, much larger starting sizes are appropriate. In contrast, when the molecular model approaches completeness the starting size of the atomic radii should be reduced. After the mask has been created, it can be expanded or reduced, and separate clouds of mask points can be removed and cavities can be filled.

In the absence of an atomic model (including the inability to create one), masks can be created from the density map itself. Firstly, the skeleton atoms are derived from the density map. The asymmetric unit of the crystal is then chosen by displaying and editing the density skeleton. (Editing means breaking and creating bonds between skeleton atoms.) After the asymmetric unit has been established, the skeleton is further subdivided into regions related by the NCS. We applied this procedure for the first time during structure determination of *N*-carbamoylsarcosine amidohydrolase (Romão *et al.*, 1992 ▶).

When partial models are already available, it is simplest to expand the mask by interactive positioning of α-helices or β-­strands into the existing molecular model. The occupancy of their atoms should be set to zero to confine their use to mask generation and to exclude them from phasing.

#### 2.1.3. Map averaging: solvent flattening

After the generation of the unit cell from the maps of NCS subunits, the unmasked regions of the density remain empty. The density in this region can be flattened (Wang, 1985 ▶), flipped (Abrahams & Leslie, 1996 ▶), shifted, scaled or modified in any way that the user desires.

In the absence of NCS, the density-modification procedure reduces to solvent flattening. When no model is available, the molecular mask can be assigned from the histogram of the scoring map (Abrahams & Leslie, 1996 ▶; Leslie, 1987 ▶). The regions above the threshold, the denser parts of the map, are considered to be molecular regions, whereas the rest is treated as the solvent region.

#### 2.1.4. Map averaging: deriving density-superimposition parameters

The geometric parameters for the transformation of equivalent density regions within the NCS group can be obtained by the superimposition of equivalent molecular structures or of equivalent regions of the electron-density maps themselves. These parameters consist of the rotation matrix and the translation vector.

The simplest way to obtain the transformation parameters between equivalent regions is by superimposition of molecular models. It is also easy to envision that heavy atoms can represent a molecular model. The heavy atoms can be superimposed as soon as their positions are assigned to local subunits. From the model, parameters are derived by an r.m.s. fitting procedure. *MAIN* uses segment identifiers (segment names) to address the local subunits of the model. Clearly, the segment identifiers (names) must be assigned before the r.m.s. fitting procedures can be applied.

In the case of proper symmetry, the superimposition parameters have less freedom. When the positions of the molecules are related by an *n*-fold rotation axis, the *n*-fold symmetry of the axis should be kept fixed (360°/*n*) for the entire group of superimposition operators. This is achieved by performing the r.m.s. fit optimization in polar angle space. For icosahedral symmetry, however, all superimposition matrices are fixed and the centre of rotation becomes the gravity centre of the multimer (often the origin of the unit cell).

When the molecular model cannot be built and the heavy-atom positions do not reveal any NCS, then the superimposition operators must be derived from the density map itself. However, the phases should be of sufficient quality to reveal the molecular masks, otherwise the rotational and translational components cannot be searched undisturbed by the crystal symmetry. To identify the masks in *MAIN*, they must be marked (using interactive map skeletonization and assignment). In this process, exploitation of the data provided by the self-rotation function of the Patterson map may prove to be useful. *MAIN* enables the optimization of superimpositions of the local map by maximizing the correlation between the background map and the density values of rotated and translated points of the superimposed map. This procedure was developed in the structure determination of *N*-­carbamoylsarcosine amidohydrolase, for which it is also documented (Romão *et al.*, 1992 ▶). Superimposition of maps using the molecular-replacement software *AMoRe* (Navaza, 1994 ▶) was applied for the first time for the alkaline protease from *Pseudomonas aeruginosa* (Baumann *et al.*, 1993 ▶). (One can also use programs such as *Phaser*; McCoy *et al.*, 2007 ▶). In such cases, density within the mask must be transferred to a unit cell large enough to prevent cross-correlation based on the ‘crystal’ symmetry. These parameters should then be refined using real-space density grid point superimposition, as described by Ploom and coworkers for the structure determination of dihydroneopterin triphosphate epimerase (Ploom *et al.*, 1999 ▶). As soon as an improved density map enables the first fragments of the structure to be built, the positioning of the resulting model should be refined against the background electron-density map using the NCS restraints followed by the updated superimposition parameters. These two steps should be repeated each time after the model is expanded.

#### 2.1.5. Map averaging: density averaging

In *MAIN*, density is averaged at each masked grid point. Using rotational and translational operators, the coordinates of the grid points are transformed to equivalent regions in the map. The density at the transformed position is obtained by linear interpolation from the surrounding eight grid points using the 8–4–2–1 point interpolation scheme. After addition, the density within each molecular mask is averaged (scaled). When necessary, the density can also be shifted or otherwise manipulated. The resulting density points within each masked region are used to fill the unit cell by applying the crystal symmetry operators.

Density in regions of space allocated to molecules that are not present in multiple copies is also used to fill the unit cell using the crystal symmetry operators.

#### 2.1.6. Map averaging: cycling through reciprocal space

After each density-averaging cycle has been completed, the unit cell is generated and the solvent region is adjusted (either flattened or flipped). Typically, the resulting map is cycled through reciprocal space to calculate 2*F*_obs_ − *F*_model_ or *F*_obs_ maps, which can be phase-combined. *F*_model_ contains contributions from the atomic model (*F*_calc_) and molecular mask, sometimes termed the molecular envelope (*F*_env_). However, when the resulting density map after the Fourier transform appears worse than after real-space averaging, cycling through reciprocal space should be postponed until the molecular masks, superimposition parameters or both are sufficiently improved to result in an improved Fourier-transformed map.

The *F*_obs_ − *F*_model_ and Patterson maps can also be averaged; however, their Fourier transforms do not make sense. The *F*_obs_ − *F*_model_ difference maps may substantially improve the density corresponding to a ligand or solvent molecule, whereas averaging of the Patterson map can help to refine the rotation axis and angle and to reveal the number of subunits present, as in the case of the proteasome (Löwe *et al.*, 1995 ▶), for which the electron-microscopy data and the unmodified Patterson map could not clearly differentiate between sixfold and sevenfold rotation axes.

### 2.2. Map skeletonization

The map skeleton is a useful feature to assess the map interpretability and to help a user to decide whether the model should enter model building, whether the case should be submitted to further density modification or whether additional experimental data should be acquired to successfully determine the structure.

**Figure 3:**
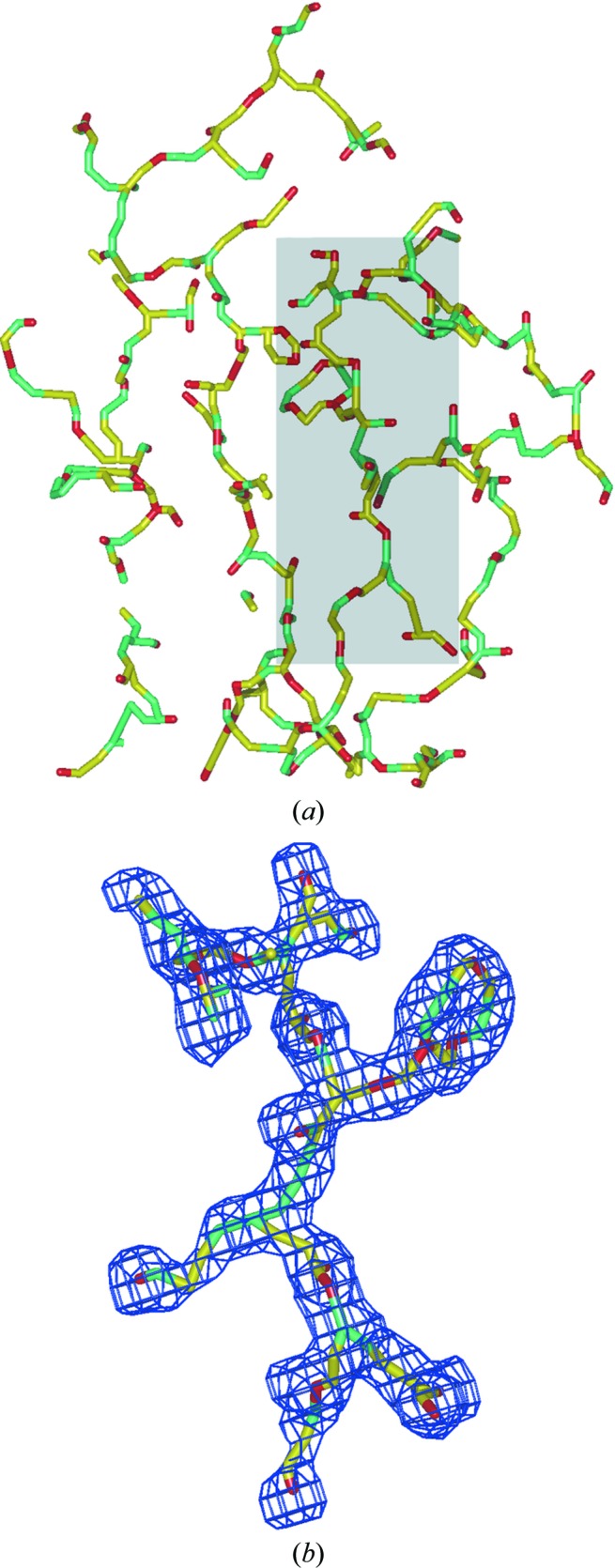
Map skeleton. The skeleton is shown as a stick model with colour codes corresponding to roles: the map extreme points are shown in red, the saddle points in cyan and the connections between them in yellow. (*a*) shows the skeleton of a 2*F*_obs_ − *F*_model_ map around a molecule, whereas (*b*) shows a portion of the region from the shadowed frame of the skeleton together with the map from which it was generated. *POV-Ray* was used to render the image.

The skeletonization procedure as incorporated in *MAIN* is based on the idea of Swanson (1994 ▶) of searching for local extremes and finding the saddle points between them. The difference in implementation is that the procedure does not include any sorting. The procedure involves three passes through the density map. In the first pass, each grid point with density within the specified range is assigned a pointer that points to the neighbouring point with the highest density. The extreme is the point that points to itself because it has the highest density value among all neighbours. The second pass traverses from the extreme points ‘downhill’ in the reverse direction to the pointers to assign the grid points pointing ‘uphill’ to the same local extreme. The third pass finds the border points between each pair of two neighbouring extreme ‘hill’ areas. The two neighbouring points with the highest density between each pair of touching density hills become the saddle points. The extreme and saddle points are transformed into atoms connected by covalent bonds either directly or along the ‘uphill’ path. The resulting ‘atomic’ object can be displayed as a molecular image (Fig. 3 ▶) and edited.

### 2.3. Model building

Model building in *MAIN* includes the creation and modification of the topology of molecules (connectivity, atoms and residue records) and their placement into the desired positions in the electron-density map.

The provided topology library entries contain a description of amino-acid and nucleic acid residues using the parameter sets of Engh & Huber (1991 ▶) and Parkinson *et al.* (1996 ▶), respectively. The most common ions are available in the default distribution, whereas for other ions the topology and geometry restraints server (Andrejašič *et al.*, 2008 ▶) compiled from the Cambridge Structural Database (Allen *et al.*, 1979 ▶; Allen, 2002 ▶) is used. Alternatively, residue entries can be imported in *CNS* file format.

Once a topology entry and the corresponding geometry restraint parameters have been read into *MAIN*, the residues can be created and manipulated using model-building and energy-minimization tools. It should be noted that by default *MAIN* uses explicit hydrophilic H atoms which, in combination with the electrostatic energy term, provide a means of stabilizing electrostatically favourable contacts in addition to the conformations of main and side chains. H atoms assist in the assignment of hydrogen-bonding patterns and are used to identify and assign secondary-structure patterns.

In *MAIN*, *de novo* molecular models can be built, extended and rebuilt. The order of the following subsections is intentionally reversed because most users begin with model rebuilding, as the majority of initial models are currently automatically generated or result from a molecular-replacement solution.

#### 2.3.1. Model rebuilding

Efficient model rebuilding is achieved by combining the automated and manual model-rebuilding tools with energy minimization. There are tools that correct distorted regions of the structure to a reasonable geometry, tools that guide the atoms into the density by searching for the best fit to the electron-density maps and tools that combine the chemistry and the electron-density terms in energy minimization.

The automated tools act on the selection, which is either the last residue or one of the groups of residues (part of a chain, covalently attached atoms, segment ID, an entire working model or user-defined), whereas the manual tools operate on clicked atoms, residues or selections.

There are tools that correct the geometry of main and side chains. The FIX_PEPT item rebuilds the geometry of peptide-bond atoms into the ideal *trans* conformation, the EXT_SIDE item finds the rotamer with the most extended conformation (including as many *trans* conformations as possible) and the FIX_SIDE item finds the closest rotamer to the current position of the side-chain atoms.

The flipping tools FLIP_PEP and FLIP_SIDE change the orientation of the peptide bonds or of the last branch of a side chain by 180°. Side-chain flipping is used in the hydrogen-bonding optimization routine, which flips the side chains while maximizing the hydrogen bonding.

Peptide bonds and side chains can be fitted into electron density by a search about every rotatable bond. The search factors in the neighbouring main chain and C^β^ atoms, the secondary structure and the electron density. Alternatively, a stretch of covalent bonds between two clicked atoms can be rotated about each rotatable bond and a selected group of atoms or residues can be rotated and translated into more appropriate positions.

Automated tools can be combined with the manual tools. Manual geometry changes can be imposed on any molecular structure presuming that its atoms are displayed and that connectivity (covalent bonds) exists between them. Individual or groups of atoms can be translated and rotated about the *x*, *y* and *z* directions, rotated about bonds (chain rotations) and internal coordinates. Any of these functions can be combined in any order. For example, rotation and translation can be imposed on the position of the entire ligand molecule and combined with rotations about bonds, including rotations about the same bond in both directions. Additionally, it is possible to monitor an essentially unlimited number of distances, angles and dihedral angles, including the position of a residue, in the Ramachandran plot.

After model rebuilding, energy minimization using the real-space target should follow.

In particular, model rebuilding is accelerated by several single-key shortcuts that include side-chain fitting, peptide-bond fitting, fragment fitting, minimization and a few others, which when used in combination enable rapid rebuilding of the local structure. When these tools are combined with validation tools, local errors and energy hotspots can quickly be identified and corrected.

#### 2.3.2. Model extension

By extension of a molecular model, I mean that residues or atoms will be attached to the current model. The residue network can grow by interpretation of the side-chain and main-chain atoms and the addition of solvent and ion molecules and other ligands.

The trace side-chain tool (TRC_SIDE) enables the recognition of amino-acid side chains by screening through the list of residues. This tool finds the residue topology that best corresponds to the electron-density map at the displayed contour level. (Topologically equivalent pairs of residues such as Asp and Asn, Glu and Gln, and Val and Thr are not differentiated.) Residues can also be changed and extended by explicit user specification of residue names. Once a sufficient portion of the sequence has been recognized, the sequence can automatically be extended to the stretch of neighbouring residues by accessing the sequence information from the protein-sequence file.

The solvent-generation tool is composed of the configuration macro, which populates the corresponding peaks with solvent molecules from the user-specified height of the peaks in the *F*_obs_ − *F*_model_ and 2*F*_obs_ − *F*_model_ maps and the suitability of the chemical environment. The development of this procedure began with my first crystallographic task, in which I had to complete the refinement of the structure of thrombin (Bode *et al.*, 1992 ▶). Solvent molecules can also be added manually or *via* a semi-automated procedure which guides the user sequentially through a sorted list of peaks (sorted by height). At each position the user can decide to add a solvent molecule or skip to the next.

Ions and other ligands must be placed manually.

#### 2.3.3. Initial model building

**Figure 4:**
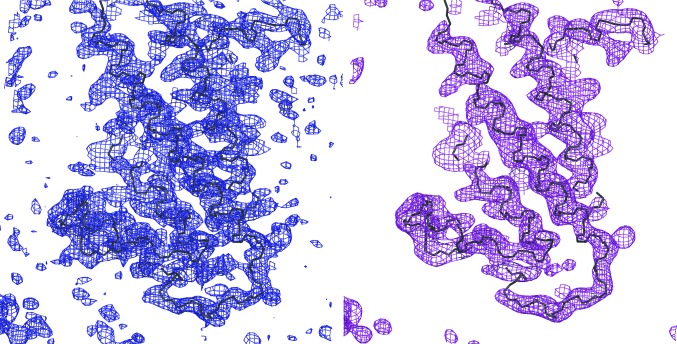
Score-map demonstration. On the left is a detailed view of a region of MAD electron density (blue) superimposed on the chain trace of the final structure (black). On the right is the identical region of the score map generated with a 2.6 Å sphere radius (pink) and superimposed on the chain trace (black). The example of cytochrome *c* oxidase (Soulimane *et al.*, 2000 ▶) was used to generate this figure. *POV-Ray* was used to render the image.

Once an electron-density map of sufficient quality has been generated by the phasing tools (including density modification), atomic models can be built into an electron-density map. The chain-trace layout can be interpreted automatically with the assistance of the TRACE tools or manually. (Because the TRACE tools are a work in progress, they are not described here.) For recognition of the secondary-structure elements, it is helpful to display a skeleton of the map and to calculate the score map for the electron density using a smaller sphere radius (2.0–3.6 Å) than that used to generate the molecular mask (>10 Å; Leslie, 1987 ▶; Fig. 4 ▶).

The tools for manual interaction with the conformation and position of the molecular model are certainly one of the highlights of *MAIN*. In these cases, the user can build stretches of secondary-structure elements in the maps and optimize their geometry and density fit by real-space minimization procedures. The secondary-structure table includes all basic secondary-structure elements and those less commonly used, such as polyproline and collagen folds. Type I, II, III and γ turns and their inverses are also available. The folding elements can be assigned in forward or backward directions to any size of chain segment. The user can browse through them by clicking until a reasonable starting point is found. The chain-geometry functions can also be called when the manually driven positional changes of the model are active.

The user can obtain the most out of *MAIN* when manually and automatically driven geometry changes of a model are combined with energy-minimization procedures. In particular, at lower resolutions the hydrogen-bonding networks should be used to restrain the structure to near-ideal secondary-structure geometry.

### 2.4. Energy calculations and minimization

The energy calculations are based on the topology libraries and corresponding force-field parameters. Energy minimization is based on the conjugate-gradient approach as described by Press *et al.* (1992 ▶). *MAIN* uses the standard bonding (bonding distances and angles, dihedral and improper angles) and nonbonding (van der Waals and electrostatics) energy terms as used in programs such as *X-PLOR* and its successor *CNS* (Brunger, 2007 ▶; Brünger, 1992 ▶). In addition, there are also the real-space electron-density map term and restraints such as dihedral, NCS-pair and hydrogen-bond restraints, which are briefly described here.

(i) The density term pulls atoms along the gradient of the local density. The second-order polynomial is fitted from four points of electron density along each of the three directions of the grid (*a*, *b*, *c*). Density at these four points is calculated by linear interpolation from the four grid points of the closest cross-section. The second-order polynomial is used to calculate the first-order derivatives. The density term should be used throughout the model-building sessions. At the beginning of each minimization cycle, the density scale is adjusted to the distortions of the geometry. The scale is adjusted to equilibrate the model at the target r.m.s. deviation of the bonding term. The default bonding deviation target is set to 0.02 Å and can be changed by the user.
(ii) The dihedral restraints enforce conformations. These restraints must be specified explicitly by the user. Recently, hydrogen-bond restraints have replaced dihedral angle restraints. These restraints have the advantage of being calculated on the fly, and their list can be edited by clicking the atoms.
(iii) The hydrogen-bonding terms used in *MAIN* are distance restraints. These terms pull H atoms (donors) towards their acceptors. Their major purpose is to stabilize and regularize secondary structures. At lower resolutions, where the electron density does not enable resolution of the positions of carbonyl groups, use of the hydrogen-bonding terms becomes mandatory to ensure building of regular secondary-structure elements such as α-helices and β-sheets. Hydrogen bonds can be assigned by the secondary-structure recognition tool, calculated from interatomic distances and specified manually.
(iv) NCS restraints pull molecular models of the NCS group towards the equivalent atoms of their superimposed average structure.
(v) The pair constraints pull atoms against each other toward a specified target distance with a harmonic force.

An important tool to increase the convergence radii during energy minimization is kicking. In *MAIN*, atoms can be kicked (displaced randomly) in the *x*, *y* and *z* coordinates from their current positions. Kicking is a computationally inexpensive method to overcome local energy minima and aids in reducing model bias. However, larger shifts (beyond 1.0 Å) should only be applied locally in areas directly supervised by the user.

When using the *MAIN* model-building and rebuilding tools and validation, there is no real need for explicit database support of the geometry beyond the force field. Use of these parameters in energy minimization and structure validation are a sufficient warrant of reasonable geometry.

### 2.5. Structure refinement

In contrast to minimization of the local structure, refinement in *MAIN* is understood as fitting of the entire structure against the experimental data. Positions, isotropic temperature factors and occupancies of atoms can be refined. Refinement of the atomic anisotropic displacement factors is currently not supported. The fitting procedure is equivalent to the energy-minimization procedure described in §[Sec sec2.4]2.4. The chemical energy terms are identical. The difference between the two is in the crystallographic target selection. In energy minimization performed during model building only the real-space density target is applied, whereas in refinement in addition to the real-space term several targets can be used such as the electron-density map, which can be updated or not during refinement, and reciprocal-space targets using least-squares and maximum-likelihood functions (Lunin *et al.*, 2002 ▶). Kicking of the atomic coordinates and *B* factors is used to increase the convergence radius of refinement. Kicking can be introduced in cycles, in which each begins with an identical or linearly decreased structure perturbation.

During the refinement, experimental and chemical energy terms can be combined with the NCS restraints. The NCS restraints can be imposed between various molecules regardless of their origin. In addition to the same crystal, they may arise from different crystal forms or from background models determined at higher resolution. (The background models are not refined; they only serve as the target.) To my knowledge, the structure of human procathepsin B was the first reported case of a macromolecular structure that was refined simul­taneously in two crystal forms (Turk *et al.*, 1996 ▶).

The refinement macros are configured *via* command-line or GUI interfaces. *MAIN* is open to cooperation with other refinement programs by exporting the coordinate file. The structure can also be submitted to *REFMAC* for refinement directly from the *MAIN* session (Murshudov *et al.*, 1997 ▶, 2011 ▶). The interface is directly configurable from the *MAIN* ‘REFINE’ macro.

### 2.6. Structure validation

The structure-validation tools assist the user in tracing and fixing local errors in the structure.

The simplest interactive structure analysis is to keep the images of related molecules superimposed on the currently built model and to investigate the differences between them by visual comparison.

A rather more complex validation with multiple choices is provided through the GUI configurable tools accessible *via* GEN_LIST on the BLD_RESI and VALIDATE topical pages. These tools highlight potentially problematic areas in the model. The general idea is that the outliers (3σ by default) for a number of criteria are found and sorted from the highest to the lowest deviation, and the top 50 (by default) are then stored for inspection into the list of centres. The centres are located at residues, atoms or other positions in space, such as difference map peaks. The single key press ‘g’ for ‘go’ moves the centre to the next atom/position on the screen and ‘G’ performs a move to the previous atom/position. A user can choose to make a correction or to move to the next position.

Validation can be performed for criteria such as packing, bonding-term deviations, *B* values, the fitting of residues and atoms to the electron-density map and the position of peaks in electron-density maps.

(i) High deviations of bonds and angles from their target values reveal areas in which a strained conformation should be released by movement or minimization.
(ii) Packing problems indicate errors in positioning. To resolve this problem, atoms or residues should be moved manually. When nonbonding interactions are validated, the presence of H atoms is mandatory as only their presence assures the correctness of the electrostatic energy calculation. Alternatively, for main-chain packing Ramachandran plots may be displayed. The Ramachandran plot monitor enables an inexperienced user to move a polypeptide-chain conformation into the allowed regions of a Ramachandran plot while rotating the model about the ϕ and ψ angles and monitoring their position in the Ramachandran plot.
(iii) Optimization of asymmetric termini of the side chains of residues such as Asn, Gln, His and Thr also requires the use of H atoms. The hydrogen-bonding network procedure checks for the most appropriate positioning of these side chains by flipping them by 180° and chooses the most appropriate positions (Turk & Turk, 2010 ▶).
(iv) Density-peak analysis can guide a user through density peaks that may require the positioning of additional solvent molecules or suggest corrections of incorrectly placed models and expose multiple conformations.

Alternatively, one can also use colour to visualize energy or *B*-value hotspots in the structure.

### 2.7. Superimposition of molecular models

Explicit matching from pair definition, sequence ID matching and distance calculation between C^α^ atoms are used. In the case of lower structure similarity, *j3D_CE* (Shindyalov & Bourne, 1998 ▶) and *jFATCAT* (Ye & Godzik, 2003 ▶) can be used in a locally installed Java version. One can also use the structure–sequence alignment table produced by *STRAP* (Gille & Frömmel, 2001 ▶) and *ClustalW* (Thompson *et al.*, 1994 ▶) in the ‘msf’ and ‘aln’ formats, respectively. These file formats are transformed by the seq_align_to_main.pl script into a list of C^α^-atom pairs used in the *MAIN* r.m.s. fit-guided superimposition.

## 3. Two cases

Here, two recent cases are described in which the interactive use of *MAIN* was crucial for successful crystal structure determination. Both cases required manual *de novo* tracing of the molecules, and in both cases fourfold NCS density averaging was exploited. The crucial concept behind the building of the structures was to postpone the introduction of model bias to the latest point possible. Both models were built to a sufficient size by exploiting the interplay between molecular masks and superimposition parameters. This strategy continued for as long as additional residues could be built. Only then were the atomic structure factors of the model under construction introduced into the structure determination in map calculations and structure refinement. The first case is based on selenomethionine phasing at rather low resolution, whereas the second case is based on molecular-replacement phasing with a known partial structure. The crucial steps in the structure solutions are described below.

### 3.1. Selenomethionine case: phasing at 3.35 Å resolution

**Figure 5:**
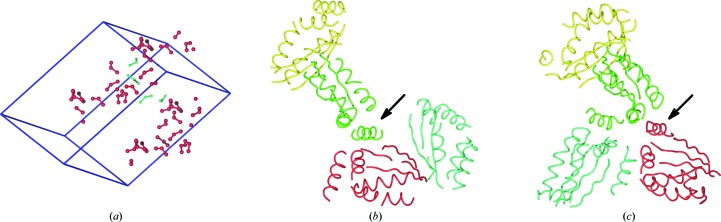
The first three stages of structure interpretation in the phasing of the selenomethionine case. *POV-Ray* was used to render the images. (*a*) The refined Se-atom positions are shown as spheres. The three Se atoms belonging to the same molecule are connected by sticks. The four clusters of Se atoms assigned to the asymmetric unit are shown in cyan and their symmetry mates are shown in red. The unit cell is shown as blue lines. (*b*) Using the assigned Se atoms, the model was built around these selenium positions. The built segments are shown as yellow, green, red and cyan chain traces. The arrow points to the misassigned α-helix. (*c*) The assignment of the helix was corrected and the cyan molecule shifted to the left into the position which relates the green, cyan and red molecules by the threefold NCS rotation axis.

The structural genomics target RPA0582, a protein of unknown function from *Rhodopseudomonas palustris*, crystallized in space group *R*3 and diffracted to 3.35 Å resolution (PDB entry 3dca; Midwest Center for Structural Genomics, unpublished work). It was phased using a selenomethiononine derivative. The structure resulting from automated model building was partially incorrect and could not be improved; therefore, it was built manually by exploiting the NCS symmetry. In Fig. 5 ▶ the first three crucial steps leading to model completion are shown. The described procedure took about a day.

(i) The minimum requirement for exploiting noncrystallographic electron-density averaging is that the molecular masks of individual molecules are defined and that the density-superimposition parameters are accurate enough to result in a map which is better than the input. As 12 heavy-atom positions were refined, they were displayed together with their symmetry mates to identify the positions which could be assigned to individual molecules. There were three methionine residues in the sequence. Therefore, three positions had to be identified which belonged to the same molecule. Luckily, the clusters were identified as groups of three selenium positions in which the central position was closer than 11 Å to the two others. (In *MAIN* bonds were calculated with an 11 Å cutoff.) From the pattern of longer and shorter distances between the three positions, the correct superimposition of the clusters was identified. It remained to choose the four clusters positioned close together. Fig. 5 ▶(*a*) shows the result. Not presented in Fig. 5 ▶(*a*) is which of the positions were retained from the original heavy-atom refinement and which of the positions were taken from symmetry-related positions. At this stage, the atoms within each cluster of Se atoms building the asymmetric unit were given unique atom names to enable their reorganization into the residue data structure and thereby enable the calculation of superimposition parameters between the four newly formed residues. These four residues were used to define the first molecular mask using large atomic radii (15 Å, if I remember correctly).
(ii) Initial electron-density averaging was performed within the given initial masks. As soon as the first two secondary-structure elements had been built, they were refined in real space using the averaged density map as the target. The segment was then multiplied and the three resulting new segments were transferred into the areas of the other three molecules by exploiting the NCS superimposition parameters. These four starting molecular segments were then refined in real space against the original heavy-atom map using the NCS constraints. (I have to stress here the following guideline. Even though the initial density-modified map, whether based on NCS averaging or not, results in a map with a better figure of merit, this may be a dead-end street. Therefore, bias of the molecular envelope has to be avoided or at least monitored by visual comparison of the original heavy-atom and the density-modified maps.) The refined model was used to update the map-superimposition parameters and to expand the molecular envelope. From here on, the molecular models were built, transferred to other molecular masks and refined against the heavy-atom and the averaged map using NCS, and the superimposition parameters were updated until nothing more could be built. Cyclic averaging was then introduced and the procedure was repeated until no more elements could be built. This stage of the model is shown in Fig. 5 ▶(*b*).
(iii) Incorrect assignment of the ‘local’ NCS unit is difficult to avoid. In Fig. 5 ▶(*b*), the helix indicated by the arrow was incorrectly positioned. By transferring it from the green to the red segment, the molecular envelope could be assigned correctly and model building could proceed. Fig. 5 ▶(*c*) also shows that by shifting the cyan segment to another position in the cell, an NCS threefold axis was identified. The protein crystallized in trimers, one related by the NCS threefold axis and the other positioned on the crystallographic threefold axis.

Subsequent steps utilized the structure-factor contribution of the model combined with the Hendrickson–Lattmann coefficients from the phases of heavy-atom refinement and density modification. These averaged maps enabled the complete model to be built and refined.

### 3.2. Molecular-replacement case by partial model

The cathepsin V–clitocypin complex crystallized in space group *P*2_1_2_1_2 and diffracted to 2.3 Å resolution (Renko *et al.*, 2010 ▶; PDB entry 3h6s). Molecular replacement positioned four cathepsin V molecules, whereas the search for positioning of the bound model of clitocypin failed; it turned out later that this was owing to the lack of an appropriate model. Autobuilding software failed to deliver promising models.

Since the asymmetric unit contained four pairs of molecules, NCS symmetry could be applied. The initial map-superimposition parameters were obtained from the four molecules of cathepsin V.

**Figure 6:**
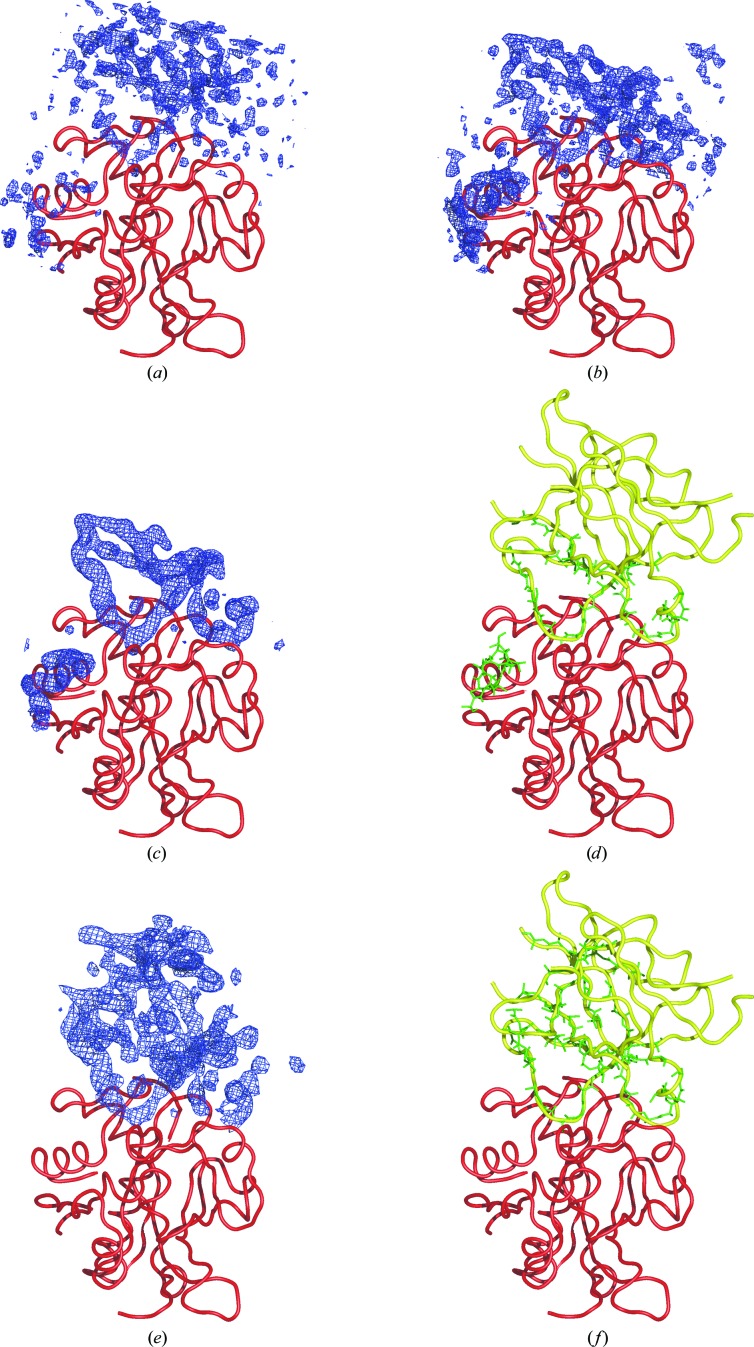
The first two stages of electron-density interpretation in the phasing of the molecular-replacement case. (*a*), (*b*), (*c*) and (*d*) show the first stage of model building. The electron-density maps shown in (*a*), (*b*), (*c*) and (*e*) are only displayed in areas that are not occupied by cathepsin V molecules. In (*a*), (*b*) and (*c*) the 2*F*_obs_ − *F*_model_ maximum-likelihood weighted map, the real-space averaged map from (*a*) and the score-averaged map from (*b*) rendered using a 2.2 Å sphere are shown, respectively. In (*d*) the molecular model (shown in green) built using these maps is shown on the background of the chain trace of the final structure of clitocypin. (*e*) and (*f*) show the second stage of model building. In (*e*), which is equivalent to (*c*), the score map of averaged density is shown. (*f*) is equivalent to (*d*); the difference is that here the molecular model at the end of the second stage is shown. *POV-Ray* was used to render the images.

(i) The initial molecular masks were generated from atoms of cathepsin V molecules using large maximal atomic radii (15 Å). The maximum-likelihood weighted 2*F*_obs_ − *F*_model_ map without the use of the bulk-solvent correction (Fig. 6 ▶ *a*) indicated the possible presence of a clitocypin-binding loop within the active site of cathepsin V; however, this map was not of sufficient quality to enable reliable model building. After averaging the electron density became clearer (Fig. 6 ▶ *b*) and the chain trace became obvious in the score map (Fig. 6 ▶ *c*). Using these maps the green parts of the model binding to the active-site cleft could be built (Fig. 6 ▶ *d*). At this stage, it was also impossible to conclude to which molecule the small green region without the yellow background of the final structure belonged (Fig. 6 ▶ *d*).
(ii) The next step included real-space refinement of cathep­sin V molecules with the partial model of clitocypin included in the generation of the molecular mask for bulk-solvent correction but not contributing to the sum of the atomic structure factors. The real-space refinement used the updated 2*F*_obs_ − *F*_model_ maps combined with NCS. With the improvement brought by the updated density-averaged map resulting from the updated superimposition parameters and the growing molecular mask, the clitocypin model expanded. After several cycles of refinement, averaging and model building it became clear that the separated helical region on the right belonged to a different complex. With this setup, we were still unable to complete the clitocypin model (Fig. 6 ▶ *f*). Namely, the NCS operators between the different clitocypin molecules in the asymmetric unit were similar but not equivalent to those that superimposed the cathepsin V molecules. Therefore, the density corresponding to the more distant parts of the clitocypin structure was averaged out as long as the NCS operators obtained from cathepsin V molecules were applied (Fig. 6 ▶ *e*).

Nevertheless, the clitocypin model grew to a sufficient size that enabled its treatment as a separate NCS group independent of the cathepsin V molecules. Clitocypin segments were included in the real-space and reciprocal-space refinement and electron-density averaging from this stage on. With the help of the superimposed model of macrocypin (Renko *et al.*, 2010 ▶; PDB code 3h6q), the connectivity of the strands could finally be confirmed and the structure determination of the complex could be completed.

## 4. Performance and specifications

*MAIN* is primarily an interactive program. The requests for interactivity depend on the task performed. The response of the displayed image is interactive as long as the image transitions are continuous nonjagged movements of the objects on the screen, whereas for the computational tasks the interactive response implies that the result should appear on the screen before the user’s mind wanders away from the subject. In the performance tests, the desktop computers and laptops listed in Table 2 ▶ were used.

### 4.1. Three-dimensional perception and graphical performance

Three-dimensional perception of molecular objects and density maps is crucial for correct interpretation of a macromolecular crystal structure. Every structure determination has a point at which the insight of a crystallographer can improve the interpretation of the crystallographic data. This insight is provided by a computer-driven graphical display, for which the clarity of perception originates from two entwined and equally important constituents: the clarity of the picture and the possibility of performing modifications of its view on the screen smoothly. In order to write efficient code and achieve clear presentation, the principles and the limitations of the hardware and software underlying the graphical visualization should be analyzed. Below, the ‘secret ingredient’ of *MAIN* graphics is revealed.

**Figure 7:**
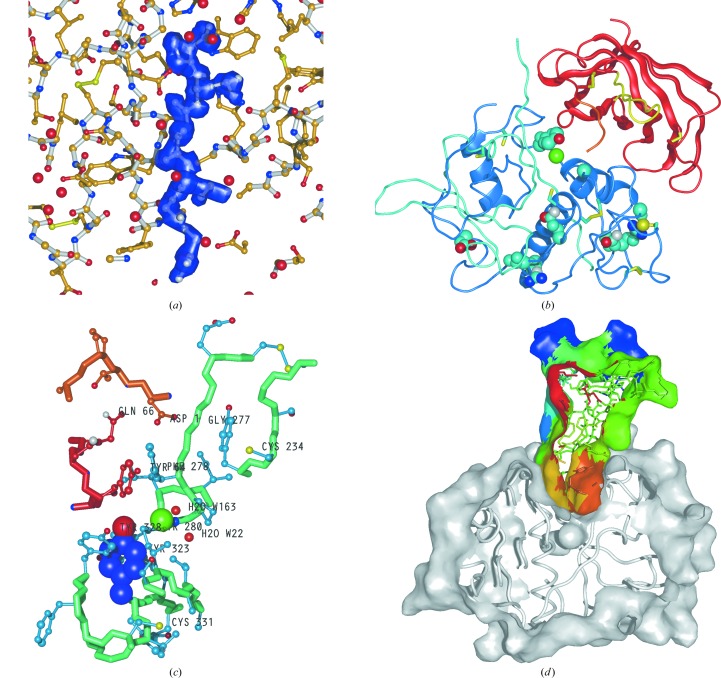
Selection of *MAIN* images. *POV-Ray* was used to render the images.

In *MAIN*, atomic models and maps can be displayed as vector lines, polygonal sticks, spheres and surfaces (Fig. 7 ▶). This section only addresses map visualizations, since they are the crucial component of the macromolecular crystal structure-determination process. (The same principles that are applied to the map presentations are also applicable to the presentations of molecular models.) It is interesting that using several graphics cards rendering of polygonal stick presentations of maps can be faster than the presentation of antialiased vector lines, which in contrast to the polygonal sticks preserve the same thickness regardless of the scale at which they are displayed. However, transitions between images of polygonal sticks are notably less smooth than transitions between images of antialiased vector lines. On the other hand, semitransparent surfaces only enable a good perception of maps in a rather slim frame in which a thin layer of residues is visible, whereas the interpretation of electron-density maps often requires insight into boxes of density which must remain within the visible frame throughout their rotation. Therefore, in macromolecular crystal structure determination, vector graphics remain the optimal way of achieving the best three-dimensional perception of molecular objects and electron-density maps. Considering vector lines, an important factor is the line thickness. Lines that are too thick are more visible and brighter; however, they disrupt the perception of smooth transitions and cover parts at the back, whereas lines that are too thin make the transition between dark and bright parts of the line presentation visible and require additional strain from a user to resolve the image. (The increased strain is a headache hazard.) The default line thickness in *MAIN* is 2.0 pixels; however, it should be optimized for the screen and for the graphics card. Other graphics options should also be explored and adjusted to achieve the best perception. (For example, when OpenGL was introduced the unfortunate default option for line presentation resulted in the appearance of disturbing bright spots at their overlapping ends, as users of the *O* program may remember.) The next important parameter is the choice of colours. This was more of an issue in the past; however, CRT displays are still in use. Namely, owing to the beam divergence on CRT displays, one should not use colours with nonzero values for all three colour components (red, green and blue). In addition, the use of all three colour components reduces the contrast between the presentation of the objects and thereby the clarity of their perception. Even though it was established decades ago, I still find the optimal choice to display the molecular objects in yellow and maps in blue with a few bits of green added on a black background.

In order to optimize the performance of the graphics objects in *MAIN*, they are precompiled into display lists and are not subjected to direct rendering *via* the immediate mode. An additional issue that is important for the efficient presentation of graphical objects is the way that they are packed into display lists. Graphical interfaces such as OpenGL libraries allow the packing of lines or triangles by adding more elements with the addition of points, also termed vertices. The graphics processors therefore deal with a smaller amount of data. By the asymptotic addition of points, the memory and work load on the graphics engines can be halved in the case of lines and reduced to a third in the case of triangles. Even more importantly, by adding the objects together the number of object lists is reduced. Because graphics processors operate on arrays, packing the data into long array lists reduces the number of steps needed to render an image. In *MAIN* the average length of the map lines in vector lists is ten. Interesting, recent tests revealed that longer lists did not affect the performance of rotation but the compilation of the lists became several times shorter. This suggests that the current Nvidia graphics libraries perform such optimizations on their own. It is expected that in the near future constraints imposed by the size of the graphics card memory will also cease to exist. Currently, these effects are very noticeable when objects with a large number of vertices are displayed.

Practically, the image response is defined by the largest difference between the transition of two consecutive images on the screen. The two images should still overlap, otherwise discontinuous jagged transitions are observed. This is clearly undesirable, as it causes headaches. In this respect, the manipulation of different parameters defining the image view and the perspective exhibit different effects on the transitions between images of displayed objects. For example, zooming transitions (in *MAIN* this is the scale at which objects are displayed) and rotations of the object exhibit completely different behaviour. Changes in the scale are more sensitive to jagged transitions as shrinking and expansion of the objects affect the whole image more than rotations, because during rotations the largest changes occur away from the centre of rotation at the periphery. It is useful to mention that the interactive changes of the image scale in *MAIN* is proportional to the scale itself, whereas the rotations are always linear increments of rotational angles. As rotations are the most often applied display modifications, they were used in the preparation of the performance indicators in Table 3 ▶. The first observation is that the image-refresh rate is limited by the display-refresh rate. As long as the graphics card is not overloaded, the screen-refresh rate defines the frequency at which the image transitions follow each other. In Table 3 ▶ the graphics performances of three Linux PCs and two Macbook Pro computers were compared on two tasks: the rotation of objects while the whole object is displayed within the screen boundaries and the time needed to prepare and submit the precompiled image to the graphics renderer. The measure of performance was in these cases the ‘elapsed’ time and not the central processor unit (CPU) time. In my impression, as long as the frequency of the image rotations remained beyond 20 Hz the image transitions appeared to be smooth. Clearly, more expensive cards can render larger maps more quickly than cheaper ones. Nevertheless, it is clear that the expensive graphics cards such as the Nvidia Quadro 5000 are primarily needed for their stereo capability and graphics quality rather than for their performance. Namely, interactive manipulation of maps containing more than 10 million vector lines are impractical. What one needs is a view into a sufficient map area with a 60 grid box or so that corresponds to about 100 000 vector lines. Such objects can be easily be rotated at 30 Hz by rather cheap graphic cards and their contouring level adjusted on the fly.

### 4.2. Computational performance

The computational performance of *MAIN* is shown in Table 4 ▶ to present how use of the *MAIN* software was close to interactive in computational tasks at the time of publication. The tests were peformed on five computers (Table 2 ▶) for three different tasks in three different cases (Table 5 ▶). The three tasks are shown in separate groups: electron-density map calculations of 2*F*_obs_ − *F*_model_ and *F*_obs_ − *F*_model_ maps, one step of cyclic density averaging (where applicable) and ten steps of conjugate-gradient refinement using the reciprocal-space maximum-likelihood target function. The map-calculation performances are presented in two parts, the first of which does not include the optimization of the tensor for overall anisotropic correction and the bulk-solvent correction parameters and the second of which includes optimization. (In *MAIN*, optimization of these parameters takes much longer than the map and structure-factor calculations themselves.) *F*_model_ combines calculation of structure factors of the atomic model and the bulk-solvent structure factors. As shown in Table 4 ▶, the size of the structure (Table 5 ▶) has a significant impact on the time needed for a procedure to complete. (The Macbooks failed to refine the proteasome structure with *MAIN* default options owing to limitations in memory allocation.) When submitting refinement of a large system, the time response stops being interactive; however, for moderate-sized systems of up to 5000 atoms one can easily wait for a refinement cycle to complete and continue with model building. Clearly, lower resolution data are computationally less demanding.

### 4.3. Compatibility with other programs

The *MAIN* software has broad applicability. *MAIN* is compatible with a variety of other programs. The input and output files of *MAIN* are all ASCII. By default, molecular models are stored in PDB format, whereas diffraction data can be exchanged through files using interfaces to the mtz format. *X-PLOR*/*CNS* map formats, *CNS* topology and parameter files can also be read.

*MAIN* has its own reciprocal refinement routines, yet programs such as *REFMAC* (Murshudov *et al.*, 2011 ▶) and *PHENIX* (Adams *et al.*, 2010 ▶) offer features such as anisotropic atomic *B*-factor refinement and TLS, which are absent in *MAIN*. (*REFMAC* refinement can be submitted from the *MAIN* interactive session, whereas for *PHENIX* refinement the saved PDB file has to be used.)

### 4.4. Technical data, origin of the name and web address

The current *MAIN* is based on the Fortran 95 standard. It uses dynamic allocation of memory such that data arrays are automatically allocated either at run time (diffraction data and maps) or at the beginning of a session (atoms and residues). Data sizes can also be reallocated at run time (atoms, hydrogen bonds, pairs, points, residues, topology entries and force-field parameters). *MAIN* is a single core executable.

Portions of the graphics and the X-window interface are written in C. *MAIN* uses OpenGL graphics libraries and runs on Linux and Mac OSX computers. Quad buffer crystal eyes stereo and side-by-side and cross-eyed stereo are supported. Mouse, dialbox and keyboard input are accepted in the image-interacting ‘dialogue’ mode. Configuration macros are written in Perl. To run the GUI interface, the Tk-Perl module must be installed. For examples, see Fig. 2 ▶ and the Supplementary Material. For the superimposition of nonidentical molecular models the *jCE*/*jFATCAT* Java bundle (Prlic *et al.*, 2010 ▶) must also be installed.

The origin of the name of the program dates back to my times in Martinsried, when I was explaining how to run the program. *MAIN* had several prompts and a dialogue mode, so in the tutorials references to them had to be established, with instructions such as ‘and then type in MAIN> the command…’. After several years *MAIN* was promoted from the internal reference to the name of the program. The entry prompt was (and still is) MAIN; however, very few users still type it in.

The *MAIN* home page (http://www-bmb.ijs.si) provides documentation, utility files, runnable cases and executable files.

## 5. Summary and conclusions

*MAIN* addresses electron-density map modifications, density-map interpretation with model building, structure refinement and structure validation. *MAIN* is oriented to present results as three-dimensional colour images that are smoothly rotatable on the computer screen. *MAIN* offers a number of unique tools such as simultaneous structure refinement of macromolecules with NCS between various crystal forms, soft group restraints for atomic *B* values, an interactive molecular-mask editor, a comprehensive list of validation tools *etc*. The major advantage of *MAIN*, however, is that all of these tools are a mouse click away. The tools can be applied immediately one after another in any order within the interactive session.

## Supplementary Material

Supplementary material file. DOI: 10.1107/S0907444913008408/dz5277sup1.pdf

## Figures and Tables

**Table 1:** Contents of topical pages The first row contains the names of the topical pages. Lists of the menu blocks of the topical pages are shown in columns below the page name.

MAPS	BLD_MAIN	BLD_RESI	MINIMIZE	VALIDATE	REFINE	SUPERIMP
NICE_SEL	NICE_SEL	NICE_SEL	NICE_SEL	NICE_SEL	SHORT_ENERGY	NICE_SEL
MAP_DO	HISTORY	HISTORY	HBONDS	HISTORY	ENERGY	DEPP_PAIR
DENS_MOD	BUILD_MAIN	BUILD_RESI_AUT	SEC_ASIGN	RAMACHAN	KICKS	SUPERIMPOSE
SET_WEIGHT	MODELER	MODELER	DEPP_MINI	ENERGY	REFINE	UN_DO
MAP_MASK	MAKE_DEL	CENTER	SHORT_ENERGY	ANALYSIS	UN_DO	
IMAGE_MAP	SECONDARY	MAKE_DEL	ENERGY			
MAP_ACTIVE	SEC_DNA	BUILD	UN_DO			
MAKE_DELETE	CENTER	AUTO_SEQ				
	UN_DO	UN_DO				

**Table 2:** Computers and graphics cards used in preparing performance tables Five systems were used to compile the tables.

Computer	Operating system	Processor	Graphics card
PC 1	PC SUSE 11.3 32 bit	AMD Phenom II X4 940	Nvidia GeForce GTX 460
PC 2	PC SUSE 11.4 64 bit	AMD Phenom II X6 1090T	Nvidia GeForce GTX 480
PC 3	PC SUSE 11.4 64 bit	Intel i7 Quad Core	Nvidia Quadro 5000
Mac 1	Macbook Pro Retina OSX 10.8	2.6 GHz Intel i7 Quad Core	Nvidia GeForce GT 650M
Mac 2	Macbook Pro 17" OSX 10.6	2.33 GHz Intel Core Duo	ATI RadeonX 1600

**Table 3:** Graphics system performances Two comparisons were performed: map rotations and map generations. — indicates tests which failed owing to memory-allocation problems. Map rotation: for map rotations the five computers had to spin the whole density box within the visible area of the graphical window. The density box was a cube of 101, 201 and 301 grid points of a map volume which corresponded to 600 000, 3 000 000 and >10 000 000 line vertices, respectively. The box sizes are actually unrealistically large. The 201 box already covered the whole proteasome molecule with the grid spacings at 1/3 of the maximal resolution. The elapsed time was recorded between a series of consecutive images rendered on the screen. The units are Hz (s^−1^). The frequencies that a human eye can cope with lie beyond 20 Hz. Within the working box sizes, which are 61 grids or less, all systems responded with the frequencies limited by the refresh rate of the display (either 60 or 120 Hz). The exception is the Apple Cinema display attached to PC 1, which could spin a few small objects at frequencies beyond 300 Hz. PC 3 had a monitor capable of quad buffer stereo attached. With the stereo mode on, the presented frequencies are halved. Map generation: for map-generation time the elapsed time was measured for vector-list preparation and its submission and compilation by the graphic libraries. The same boxes and computers were used as in the map-rotation tests. The units are s.

	Box 101, 600 000	Box 201, 3 000 000	Box 301, >10 000 000
Map rotation			
PC 1	9	3	1.5
PC 2	60	60	25
PC 3	120	120	40
Mac 1	15	4.5	—
Mac 2	30	7.5	—
Map generation			
PC 1	0.5	4	13
PC 2	0.45	3.5	11
PC 3	0.3	1.4	8
Mac 1	0.45	3.6	—
Mac 2	0.6	8	—

**Table 4:** Structures used in performance tests

Structure	Protein name	Resolution (Å)	Reflections	Non-H atoms	PDB code
AMDL	Amodytoxin L	2.6	5323	1083	3dih
CVC	Cathepsin V–clitocipin	2.2	81606	8076	3h6s
PROT	Proteasome	2.3	381290	50888	1g65

**Table 5:** Performance table Three calculations were used to prepare the performance table: map calculation, density averaging and refinement. CPU time was measured in s. Map calculation included structure-factor calculation from the atomic structure, bulk-solvent structure factors, fitting of *F*_model_ to *F*_obs_ and maximum-likelihood weighted map calculations of the 2*F*_obs_ − *F*_model_ and *F*_obs_ − *F*_model_ types. The two numbers indicate differences in the optimization of scaling. The first, shorter time corresponds to map calculations in which the bulk-solvent correction parameters and the anisotropic correction tensor from a previous calculation were used. The second time corresponds to calculations in which these parameters were optimized. An important parameter that affects the time is the precision cutoff (atom radii) at which the atomic density generation stops. In *MAIN* the default value density-cutoff value is very low (10^−7^). The user can change the precision-cutoff level to speed up the calculations, but this is not recommended. Averaging: one cycle of real-space density averaging followed by Fourier back-transform of the electron-density map. CG refinement: the structures were subjected to ten steps of conjugated-gradient refinement. Each refinement starts with structure-factor calculation and optimization of the bulk solvent and anisotropic tensor correction parameters.

	AMDL	CVC	PROT
Map calculation			
PC 1	3/4	7/40	30/120
PC 2	2/3.5	5/26	18/81
PC 3	1/3	3.5/25	15/72
Mac 1	3/5	8/23	20/95
Mac 2	5/7	10/44	41/140
Averaging			
PC 1	—	9	30
PC 2	—	6	25
PC 3	—	4	21
Mac 1	—	6	23
Mac 2	—	12	50
CG refinement			
PC 1	41	90	735
PC 2	25	63	570
PC 3	9	47	390
Mac 1	20	75	—
Mac 2	27	220	—

## Footnotes

1 Supplementary material has been deposited in the IUCr electronic archive (Reference: DZ5277). Services for accessing this material are described at the back of the journal.
