# Supplementary material for: MAIN software for density averaging, model building, structure refinement and validation
Source: Acta Crystallogr D Biol Crystallogr. 2013 Jun 13;69(Pt 8):1342–57. doi: 10.1107/S0907444913008408 (PMC3727325; doi:10.1107/S0907444913008408)
Supplement: Supplementary file 1 [file d-69-01342-sup1.pdf]

Supplementary Material

1. MAIN user interface

In MAIN, there are three levels of user interface, each corresponding to the different level of skills and commodity of the user. The first and most commonly used level is using interactive devices such as a mouse, keyboard shortcuts and dials to provide input events. Through them, the user interacts with the molecular structure and creates and modifies the working environment macros. At the second level, GUI (Graphical User Interface)-written macros can be adopted and expanded to match the needs of the user. The third level is the configuration and writing of independent commands and macros in MAIN language and Perl that provide additional functionality including the links to external programs.

1.1. Configuring a MAIN session

The working session must be configured before running MAIN. Configuration begins with the “Tk_main_config.pl” GUI (Figure 1s), which builds links between the crystallographic data (diffraction data, unit cell constants, space group), the atomic model file, the macromolecular sequence, and the topology library files and the MAIN program by writing several macros that are used at the beginning and during the MAIN session. The macros and setup can be altered and reconfigured at any stage of the crystal structure determination. To change the macros, each change of the entry fields must be followed by a click on the "doit" button. The configuration setup screen "Tk_main_config.pl" is composed of four frames as shown in Figure 1s:

- Crystal data,
- Molecular model data,
- Non-crystallographic symmetry group definitions, and
- Molecular segment name list and organization.

The text equivalents to the GUI are the "create_auto_start.pl" script, which reads in the PDB file and creates the missing topology files; the "use_*.pl" scripts, which import diffraction data; and the "create_main_config.pl" script, which handles NCS and crystal group organization. (When these scripts are used for the first time, they should be applied in the order listed here).

1.1.1. Crystal data frame

The crystal data frame displays entries of the crystallographic files and data (cell constants, space group, structure factors, resolution range) that are imported from the diffraction data files using the "import diffraction file" button. The MTZ, SCALEPACK, SHELX, and CIF file formats are recognized and processed by the "use_*-pl" scripts. When the default diffraction data columns from an “mtz” file are not appropriate, the user should change them via the "tk_use_mtz.pl" (Figure 2s) button or run the "use_mtz.pl" script directly from the terminal.

Each crystal form has its own set of entries. To differentiate between the various possible crystal forms, clearly, the cell constants and diffraction data files must be stored under different, non-default file names.

1.1.2. Molecular model data frame

The molecular model data frame displays entries with the file names of atomic model data, residue sequence data and additional topology and parameter files containing information about topology and geometric restraints of hetero compounds.

Molecular model data can be imported from the PDB file using the “import PDB” button. During the import, the missing topology and parameter files are searched in the local directory. (Their file names must contain the strings “top”, “par” or “main” and “cns”). When absent, they are generated by the PURY database server or must be provided by the user. During the import, the NCS groups can be extracted from the segment and chain names in cases of high sequence identity.

Using the "tk_read" scripts, which provide parameters for the “read.com” file directly, additional parameters such as background molecular models, maps, etc., can be provided.

1.1.3. Non-crystallographic symmetry groups frame

The NCS groups define the relationships between their members in the tasks such as electron density averaging and in model superimposition, building, and refinement. The user should define which type of NCS operation is appropriate for specific case:

- The default types “EACH” and “ONE” refer to improper averaging. “ONE” differs from “EACH” in how the averaged density is generated. For the type “EACH”, the density is averaged for the area of each segment independently, whereas for the type “ONE”, the density is averaged for one segment only and then distributed to the others by map rotations and translations. The option “ONE” is particularly useful for

systems with a substantial number of copies of an identical segment as it reduces the number of map operations from N*N to 2N.

- The type “LINK” uses the superimposition operators from the previous group. This operation is particularly useful for ligands for which initial parameters cannot be calculated from their CA and P atoms. Instead, their map superimposition parameters are derived from the macromolecule to which they associate.
- The type “WHOLE” describes the proper NCS group. In this case, a single mask encompasses all group members related by the spherical symmetry.
- The type “COMPOSIT” refers to the multiple overlapping models, including those with multiple conformations observed within only a few regions.
- The type “BACKGROUND” specifies groups, which contain the molecule that is used as the target during the refinement of a structure. This operation is particularly useful during the refinement of a structure against lower resolution data, when one would like to restrain the structure to the more reliable model determined at a higher resolution.

1.1.4. Segment names and their organization frame

MAIN uses segment names (segment IDs) to identify equivalent regions of the molecular models to which the model and density NCS restraints can be applied. In the case of high sequence identity, the NCS groups are assigned automatically from the sequence identity of chains or segment IDs in the PDB file. When a high proportion of identity is absent, the user must assign the appropriate NCS group to the segment names. Each segment should belong to one NCS group. However, to relate the NCS group members by their NCS operator(s), the group should contain at least two segment members. In the case of multi-crystal NCS, the segment names must also be assigned to the corresponding crystal form.

1.2. Running a MAIN session

After the initial setup is completed, the MAIN session can begin. The user conducts the program through MAIN windows. Interaction between a user and MAIN occurs through two interactive device-driven windows (the menu window named 'Depp pages' and the MAIN image window) and through the shell window in which command sentences can be typed.

1.2.1. MAIN image window

The largest window is the image window with the title “only a MAIN user can be cool" in which three-dimensional molecular and map objects are displayed. Much care was directed to the clarity of their presentation, the immediate response of the interactive devices (mouse and "dial box"), and the update of the image. The procedures are optimized to achieve the best performance (smooth rotations of vectorized images) and the best depth perception of 3D objects. (An important consequence is that the work with "CrystalEyes" stereo does not result in headaches, whereas in the absence of the "CrystalEyes" stereo, the side-by-side stereo can also be used). The colors of the molecular and map objects are controlled by the MAIN internal color tables. A user can, however, create customized color palettes. The background color and the contrast of the displayed objects on the background can also be changed. The contrast between the objects in the back and the front provides the depth perception, which, together with the use of clear colors on the black background, provides a relaxed view into the molecular structure. The 3D impression of the molecular images is enhanced by the smooth rotations of the objects displayed. Through these features, MAIN enables users to gain an overview of the molecular objects and insight into their details. This approach differs from the one applied in Coot (Emsley et al., 2010) in which a highlighted view is focused at the center, thereby confining the view to the structural details, which results in a less intuitive perception of 3D objects.

The clicked atoms can be used as arguments for the menu items of the DEPP PAGES and their shortcuts. The single character shortcuts enable rapid movement along the selected sequence of residues and the manipulation of their geometry by calling the automatic model rebuilding tools and energy minimization. These shortcuts are global and are accessible regardless of the “depp page” that is displayed. Additionally, there are the “control key” shortcuts, which, in combination with a key, enable the identification of a single item on the displayed page. For example, the ctrl “r” followed by “i” selects the “RE_IMAGE” item when the main “depp page” is displayed.

The control of map presentations is positioned on the maps page. Maps can be displayed as wire frame objects contoured along the cell axis layers together or individually and as a surface of polygonal areas. The values of the density map can be displayed as numbers at the corresponding grid points to support the map analysis by 3D visualization.

1.2.2. Depp pages menus

The “DEPP PAGE”s are the MAIN menu structure that enables the user to display and

manipulate molecular models and maps. (An overview of the menu blocks based on the page contents is provided in Table 1). Each menu block is composed of menu items. Each menu item submits a MAIN command. Most of the menu items call command macros and are triggered by the left mouse button click. Many of them are configurable interactively. The right mouse button click opens their GUI configuration window, such as "MAP_CALC" (Figure 2). This window contains the list of all possible command keywords, their short description, parameters with all options and their current status. To this common scheme, there are two exceptions. The first exception is the previously described "tk_main_config.pl" script (Figure 1s), which involves the initial configuration of a working session. The second exception is the "tk_re_image.pl" creation menu (Figure 3s). The image composition and its functionality require a far more condensed tabular form of presentation of options than can be provided by a simple text interpretation by a GUI, as shown in Figure 2. In addition to the molecular object names, this menu includes the text entry for atom selection, the type of representation, and the coloring style. The types of representation include molecular images that display bonds as lines and sticks, atoms as crosses and ball, molecular folds as ribbons, molecules as surfaces, labels of atom and residue names. Additionally, the interatomic distances, pair lists, hydrogen bonds, and forces acting on atoms can be displayed.

The menu items call functions that act on portions or the entire molecular structure. Each function is associated with its own set of arguments. The arguments are the last picked atoms, selections such as the last clicked residue, its surroundings, segment name, and the list of all work set segment names (IDs). The arguments remain until modified by a user action. Thereby, these arguments can be reused by a sequence of functions: a selected residue can be moved, adjusted to density by automated tools and then minimized without the need to click on its atoms again.

The main "DEPP PAGE" contains three menu blocks: the yellow menu block, which provides links to descendent, topical pages and two additional white menu blocks that address the composition of the molecular images, and the session data saves. (For advanced users, it merits to mention that the entire "DEPP PAGES" reside in macros loaded at the beginning of each MAIN interactive session. These macros can be adapted and extended to the needs and preferences of the user.) The description of the two white menu blocks follows, whereas the topical pages are discussed below in their own section “Areas of structure determination included in MAIN”.

The first white menu termed “DISPLAY” concerns the molecular display composition,

displaying the molecular objects "RE_IMAGE" and their symmetry mates "SYMMETRY" and "SYMM_CA" in addition to their rendering ("PLOT" generation). The image composition is a series of command lines stored in the macro "re_image.cmds" called by the menu item "RE_IMAGE". This macro is created by the GUI tool (Figure 3s) and enables the display of the most general combinations of molecular images. Each molecular image begins with a name followed by several command lines each defining the selection of the displayed atoms, the color, and the representation of the image model. (The GUI-created macro can be used as a template for creating custom macros). Molecular images and maps can be exported (PLOTTED) to the Raster 3D (Merrit & Bacon, 1997) and POV-Ray programs (http://www.povray.org/).

The second white menu termed “SAVES” saves the session files (coordinates, connectivity table, hydrogen bonding lists, map parameters) and the current view. Most geometry changes can be undone. However, the “UNDO” is only a single-step coordinate retrieval. To return to an earlier model state, the model coordinates should be restored from the last coordinate saved files by using the "REST_FIL" item. Additionally, there is always the option to quit the program and restart or edit the copy of the session commands saved in the “input.cop” file and rerun them.

Most topical pages contain the selection menu block that appears at the top. The selection menu items specify the contents of the selection keys "active" and "passive" which include the atoms, their extension to residues, neighborhood, residue range, segment of covalent bond network, segment IDs, and the working segment. The default selection "ADD_MODE" mode is "OFF", which always redefines the “active” and “passive” selections. When the “ADD_MODE” is "ON", these two selections can be expanded by adding new ones. Exceptions include the main page items, such as "RE_IMAGE" and "SAVE_FILE", where the configuration "tk_re_image.pl" enables a user to type in selections in the MAIN language directly, and the tools such as "REFINE" and "MAP_CALC", which require the entire structure (the list of working segment names). When the provided interactive choices are not sufficient, the user specifies the selections into the command prompt directly from the terminal window.

1.2.3. Key shortcuts to menu items

Menu items on the currently displayed menu page can be called by the sequence of three

keys. The sequence must begin with “CTRL” or “ALT” keys followed by two characters. The "CTRL" key sequence invokes the item, whereas the "ALT" key invokes its configuration GUI. The first character is matched against the first character of a menu item, whereas the second is matched against the character following the "_" sign. When the "_" is absent, then the second character is matched. In the case a unique menu item is identified, it is invoked as when clicked on. In addition to these generic shortcuts, in the code there are single key shortcuts to the functions most commonly used:

- '+' rotates the image for the stereo angle in the + direction,
- '-' rotates the image for the stereo angle in the - direction,
- 'M' toggles the image menu on and off,
- 'a' accepts geometry of currently defined active objects (“OB_ACCEP”),
- 'b' rigid body fit to map of groups of fragments ("RIG_SIDE"),
- 'c' or 'C' sets the current rotation to the last picked atom (“CENTER”),
- 'd' or 'D' drops the last picked atom from the history list (“HIS_DROP”),
- 'e' or 'E' exits the dialog mode, MINIMIZation and REFINEment (“PROMPT”),
- 'f' fix the last picked atom for MINIMIZE by removing from key "active") (“FIX_ATOM”),
- 'g' go to the next residue: a shortcut to "CENT_NEX",
- 'h' toggles menu documentation mode on and off,
- 'j' do auto fitting job: a shortcut to "FIT_AUTO",
- 'k' kick "active" atoms: a shortcut to "KICK_ACT",
- 'l' selects the last clicked residue: a shortcut to "ACT_LAST"
- 'm' minimizes the "active" key selection of atoms:: a shortcut to "MINIMIZE",
- 'n' selects the neighboring atoms: a shortcut to "ACT_NEIG",
- 'o' move the last clicked atom: a shortcut to "MOV_ATOM",
- 'p' peptide fixes: a shortcut to "FIX_PEPT" and "FIT_PEPT" together",
- 'r' or 'R' redraws the maps around the current center (“RE_MAP”),

- 's' or 'S' toggles stereo on and off (“STEREO”),
- 'u' undo the last coordinate change: a shortcut to "UN_DO",
- 'w' calls the macro “WORK_MACRO.com”, unless the character variable "WORK_MACRO" redefines it,
- 'x' move the last clicked residue: a shortcut to "MOV_RESI",
- 'z' fit side chains of residues to density: a shortcut to "FIT_SIDE",
- '1' to '0' includes or removes maps (1 to 10) from the image (since “10” is not a key, “0” is used instead).

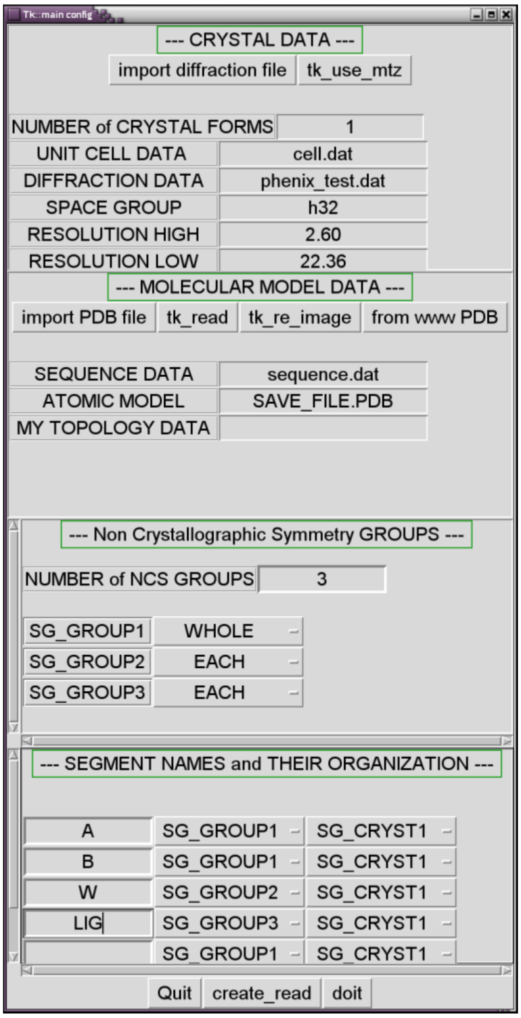

Supplementary Figure S1. Main configuration sheet. The sheet is divided into 4 frames: Crystal data, Molecular model data, Non-Crystallographic Symmetry groups and Segment names and their organization. The sunken fields accept input, whereas the buttons trigger functions (mostly import of data) and provide setup options.

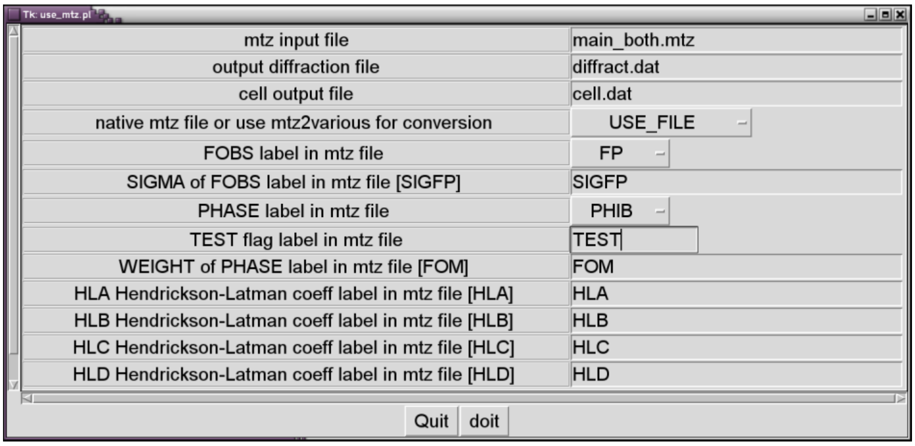

Supplementary Figure S2. MTZ input. The sunken fields accept input (input and output file names) at the top and labels for reflection data in the mtz file. The labels are read from the mtz file. When more than one option is available under a certain type of data, an option menu is created with a corresponding list.

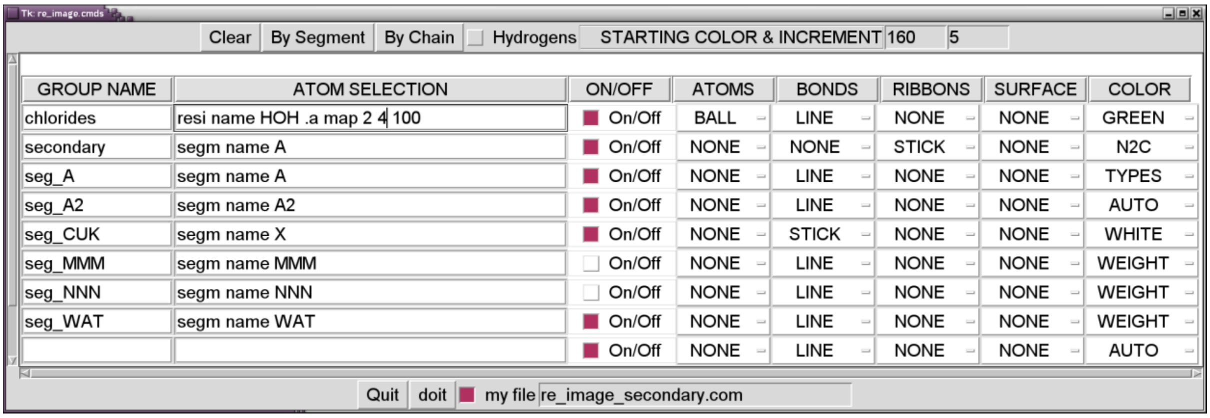

Supplementary Figure S3. “Re_image” GUI. The top bar option provides elementary options to display the molecular structure listed as either segments or chains. This option also controls the possibility to display hydrogen atoms. Each line entry in the table provides input for each group name displayed in the image window, with the selection of atoms being displayed by the representation provided with the optional list of objects. “Doit”, as everywhere, writes out the "re_image.cmds" macro, whereas the "my file" offers the possibility to redirect image generation to user created macros.

References

Emsley, P., Lohkamp, B., Scott, W. G. & Cowtan, K. (2010). *Acta Cryst.* **D66**, 486-501.

Merritt, E. A. & Bacon, D. J. (1997). *Methods Enzymol* **277**, 505-524.
